# Methodological review of NMA bias concepts provides groundwork for the development of a list of concepts for potential inclusion in a new risk of bias tool for network meta-analysis (RoB NMA Tool)

**DOI:** 10.1186/s13643-023-02388-x

**Published:** 2024-01-12

**Authors:** Carole Lunny, Areti-angeliki Veroniki, Julian P. T. Higgins, Sofia Dias, Brian Hutton, James M. Wright, Ian R. White, Penny Whiting, Andrea C. Tricco

**Affiliations:** 1 Knowledge Translation Program, Li Ka Shing Knowledge Institute, St. Michael’s Hospital, Unity Health Toronto, 209 Victoria Street, East Building, Toronto, ON M5B 1T8 Canada; 2 Cochrane Hypertension Review Group, the Therapeutics Initiative, University of British Columbia, Vancouver, Canada; 3 Population Health Sciences, Bristol Medical School, University of Bristol, Bristol, UK; 4 NIHR Bristol Biomedical Research Centre at University Hospitals Bristol and Weston NHS Foundation Trust and the University of Bristol, Bristol, UK; 5 NIHR Applied Research Collaboration West (ARC West) at University Hospitals Bristol and Weston NHS Foundation Trust, Bristol, UK; 6 Centre for Reviews and Dissemination, University of York, York, UK; 7 Ottawa Hospital Research Institute, Ottawa, Canada; 8 Ottawa University, School of Epidemiology and Public Health, Ottawa, Canada; 9 MRC Clinical Trials Unit at UCL, London, UK; 10 Dalla Lana School of Public Health & Institute of Health Policy, Management, and Evaluation, University of Toronto, Toronto, Canada; 11 Queen’s Collaboration for Health Care Quality Joanna Briggs Institute Centre of Excellence, Queen’s University, Kingston, Canada

**Keywords:** Indirect comparison, Mixed treatment comparison, Network meta-analysis, Multiple treatment comparison, Quality, Risk of bias, Critical appraisal, Tool, Checklist, Standard

## Abstract

**Introduction:**

Network meta-analyses (NMAs) have gained popularity and grown in number due to their ability to provide estimates of the comparative effectiveness of multiple treatments for the same condition. The aim of this study is to conduct a methodological review to compile a preliminary list of concepts related to bias in NMAs.

**Methods and analysis:**

We included papers that present items related to bias, reporting or methodological quality, papers assessing the quality of NMAs, or method papers. We searched MEDLINE, the Cochrane Library and unpublished literature (up to July 2020). We extracted items related to bias in NMAs. An item was excluded if it related to general systematic review quality or bias and was included in currently available tools such as ROBIS or AMSTAR 2. We reworded items, typically structured as questions, into concepts (i.e. general notions).

**Results:**

One hundred eighty-one articles were assessed in full text and 58 were included. Of these articles, 12 were tools, checklists or journal standards; 13 were guidance documents for NMAs; 27 were studies related to bias or NMA methods; and 6 were papers assessing the quality of NMAs. These studies yielded 99 items of which the majority related to general systematic review quality and biases and were therefore excluded. The 22 items we included were reworded into concepts specific to bias in NMAs.

**Conclusions:**

A list of 22 concepts was included. This list is not intended to be used to assess biases in NMAs, but to inform the development of items to be included in our tool.

**Supplementary Information:**

The online version contains supplementary material available at 10.1186/s13643-023-02388-x.

## Highlights

• Our research aimed to develop a preliminary list of concepts related to bias with the goal of developing the first tool for assessing the risk of bias in the results and conclusions of a network meta-analysis (NMA).

• We followed the methodology proposed by Whiting (2017) and Sanderson (2007) for creating systematically developed lists of quality items, as a first step in the development of a risk of bias tool for network meta-analysis (RoB NMA Tool).

• We included items related to biases in NMAs and excluded items that are equally applicable to all systematic reviews as they are covered by other tools (e.g. ROBIS, AMSTAR 2).

• Fifty-seven studies were included generating 99 items, which when screened, yielded 22 included items. These items were then reworded into concepts in preparation for a Delphi process for further vetting by external experts.

• A limitation of our study is the challenge in retrieving methods studies as methods collections are not regularly updated.

## Background

To decide the best treatment for a patient with a specific condition, healthcare providers and patients need a synthesis of the relative treatment effects for all potential treatment options [1, 2]. This comparative effectiveness synthesis would ideally involve a systematic review with network meta-analysis (NMA) of randomised controlled trials (RCTs) [3]. NMA emerged due to the limitations of standard meta-analyses to compare and rank the effectiveness of multiple treatments for the same condition [4]. Standard meta-analyses only combine effects from RCTs comparing two treatments.

NMA can help patients and their care providers choose the treatment that is most important to them based on the side effects and efficacy of all treatments. For example, Li et al. recently showed that prostaglandins would have been identified 7 years earlier as the most effective drug class in lowering intraocular pressure for open-angle glaucoma if an NMA had been performed at that time [5]. Recent empirical research also showed that NMA was 20% more likely to provide strong evidence of treatment differences compared with standard meta-analysis, and NMA provided strong evidence 4 years earlier than standard meta-analysis (because head-to-head RCTs had not been conducted that would have provided “direct” evidence) [6].

For a practicing healthcare provider, researcher or policymaker, deciding whether to believe the results from a single NMA or to choose amongst conflicting NMAs, is difficult without a tool to assess the risk of bias. An empirical evaluation identified 28 NMAs on treatment for rheumatoid arthritis [7] and found considerable discrepancies across data extracted and risk of bias assessments of included RCTs and assessment of heterogeneity. In addition, different network configurations were possible due to the different grouping of interventions considered and how they might have been merged or split into different nodes. Concerns with each of these issues leave healthcare providers and policy makers with uncertainty as to which of the biologics has the greatest treatment effect [7, 8].

Tools are available for most study designs to make quality assessment easier. For example, the Methodological Expectations of Cochrane Intervention Reviews [9] is a guideline which outlines the methods that should be followed when authors are conducting a systematic review. The ROBIS (Risk Of Bias In Systematic Reviews) [10] tool can be used by stakeholders to assess the risk of bias in systematic reviews with standard meta-analysis. Biases at the systematic review level include publication bias (e.g. where studies are missing from the published literature because they did not report statistically significant results) and selective reporting of outcomes (e.g. where outcomes did not reach a high level of magnitude or the desired direction of effect and are not reported in the published trial) or analyses. The consequence of selective reporting is that the published literature is strongly biased and will substantially overestimate or underestimate effects and associations.

The only way to deal with the problems plaguing medical science is a combined effort by researchers, editors and funding bodies to publish all science without bias and improve the quality of research that reaches publication. This cannot be done without a tool to evaluate the limitations in the way in which the NMA was planned, analysed and presented, including the way in which the evidence was assembled. If inappropriate NMA methods are used, the validity of the findings could be compromised, and decision makers will not know whether to trust the NMA results and conclusions [11–13].

Our proposed risk of bias (RoB) NMA tool will allow decision makers (defined as an individual or group who has an interest in, or affected by, health- and healthcare-related research) to assess the biases in an NMA. Our proposed RoB NMA tool is not targeted at authors of NMAs, as it does not outline how to conduct an NMA. It is targeted at decision makers such as healthcare providers, policymakers and physiotherapists, or journal peer reviewers who want to determine if the results of an NMA can be trusted to be at low risk of bias.

Checklists and tools with different aims exist to appraise NMAs, including for example, the PRISMA-NMA (PRISMA statement extension for reviews incorporating NMA, 2014) [14], used when writing up the results of an NMA, or the ISPOR (International Society for Pharmacoeconomics and Outcomes Research; [15]) checklist, used by researchers when conducting an NMA (Table 1). These review-level tools are not to be confused with tools to assess the individual primary studies included in systematic reviews (e.g. Cochrane risk of bias tool for randomised controlled trials [16]).

**Table 1:** Tools and checklists to aid in systematic review conduct and to assess the reporting, quality of conduct or the risk of bias in a review

Tool purpose	Examples of tools or checklists	Description of an example tool	Targeted users	Available tool for reviews with NMA
Guidance for conducting systematic reviews	MECIR [9]	Detailed guidance for the conduct of systematic reviews of interventions, diagnostic test accuracy, individual patient data, public health and health promotion	• Review authors • Journal editors	No
Assess the quality of conduct of reviews	AMSTAR-2 [17, 18], OQAQ [19]	AMSTAR-2 is a critical appraisal tool to assess the conduct of intervention reviews including RCTs The 1991 Overview Quality Assessment Questionnaire (OQAQ) is a methodological quality of conduct checklist	• Review authors • Decision makers	No
Assess the risk of bias in published reviews	ROBIS [10]	ROBIS is a tool for assessing the risk of bias in reviews. It is aimed at four broad categories of reviews mainly within health care settings: interventions, diagnosis, prognosis and etiology	• Decision makers	Not at present, but in the process **RoB NMA tool**
Assess the certainty in the evidence and the strength of recommendations in health care	GRADE [20]	The GRADE working group defined the certainty of a body of evidence as the extent to which one can be confident that a pooled effect estimate is close to the true effect of the intervention. Five domains were assessed: risk of bias, inconsistency, indirectness, imprecision and publication bias	• Review authors	GRADE-NMA [21, 22], CINeMA, [23], Threshold method [24]
Guidelines for the complete reporting of published reviews	PRISMA Update [25]	PRISMA focuses on the reporting of already published reviews evaluating RCTs of interventions. PRISMA can determine whether a review is well described and transparently reported	• Decision makers • Journal editors	PRISMA-NMA [14], ISPOR [15]

*AMSTAR-2* A Measurement Tool to Assess Systematic Reviews 2, *CINeMA* Confidence in Network Meta-Analysis, *GRADE* Grading of Recommendations Assessment, Development and Evaluation, *GRADE-NMA* Grading of Recommendations Assessment, Development and Evaluation for Network Meta-Analysis, *ISPOR* International Society for Pharmacoeconomics and Outcomes Research, *MECIR* Methodological Expectations of Cochrane Intervention Reviews, *OQAQ* Overview Quality Assessment Questionnaire, *PRISMA* Preferred Reporting Items for Systematic Reviews and Meta-Analyses, RCT randomised controlled trial, *ROBIS* Risk Of Bias In Systematic Reviews

Guidance on how to develop quality and risk of bias tools has been proposed by Moher [26] and Whiting [27], and one of their first recommended steps is to create a systematically developed list of bias items. Such a list of items has been created by Page et al. [28] when updating the PRISMA 2020 checklist [25]. However, there has been no attempt to comprehensively identify items from NMA quality tools, checklists and scales, which would provide a useful item bank for a proposed risk of bias tool for NMAs (RoB NMA tool), or those wishing to update existing tools or standards for NMAs. The aim of this study is to conduct a methodological review to compile a preliminary list of concepts related to bias in NMAs. The list is not intended to be used to assess biases in NMAs, but to inform the development of items to be included in our tool.

## Methods

### Management, gGuidance and protocol

A steering committee of nine individuals was convened and comprised of eight experts in NMA, tool development and evidence synthesis methodology, as well as one clinician. The steering group is responsible for the management of the project and has executive power over all decisions related to the new tool.

A methodological review is where evidence on a given methods topic is systematically identified, extracted and synthesised (e.g. Song [29] and Page [28]). We followed the methodology proposed by Whiting [27], Sanderson [30], and Page [28] as previously discussed. We published our study protocol in BMJ Open [31] and present all data on the Open Science Framework at https://osf.io/f2b5j/.

We adopt a broad definition of an NMA as a review that aims to, or intends to, synthesise simultaneously the evidence from multiple primary studies investigating more than two health care interventions of interest. We also considered in our definition the cases when multiple treatments are intended to be compared in an NMA but then the assumptions are found to be violated (e.g. studies are too heterogeneous to combine), and an NMA is not feasible. Our RoB NMA tool will aim to address the degree to which the methods lead to the risk of bias in both the NMA’s results and the authors’ conclusions.

### Paper eligibility criteria

We included papers describing instruments (i.e. domain-based tools, checklists, scales). A tool is defined as any structured instrument aimed at aiding the user to assess quality or susceptibility to bias [30]. Domain-based tools are designed to assess the risk of bias or quality within specific domains [32]. To be defined as a checklist or questionnaire, it had to include multiple questions, but without the intention to ascribe a numerical score to each response or to calculate a summary score [32]. To be defined as a scale, a numeric score was ascribed to each item and a summary score was calculated [33].

We also include methods papers and journal editorial standards that present items related to bias, reporting or the methodological quality of NMAs. We also included papers that *assessed* the methodological quality of a sample of NMAs.

#### Inclusion criteria

I. Papers describing methods relating to methodological quality, bias or reporting in NMAs of interventions
II. Papers or reports describing journal editorial standards for NMAs (e.g. comparable to the Cochrane MeCIR [methodological standards for the conduct of new Cochrane Intervention Reviews] standards [9])
III. Papers examining quality (or risk of bias) used in a sample of NMAs of interventions (e.g. Chambers 2015 [34]) using criteria that focus specifically on aspects of NMAs not just on general aspects of systematic reviews
IV. Guidance (e.g. handbooks and guidelines) for undertaking NMAs of interventions
V. Commentaries or editorials that discuss methods for NMAs of interventions

#### Exclusion criteria

I. Papers describing instruments that only assess general aspects of reviews without focusing specifically on NMAs (e.g. AMSTAR [18], AMSTAR 2 [17] or ROBIS [10]).

Papers with any publication status and written in any language were included. If we identified a systematic review of studies that would themselves be eligible for this review, we used the results of the review and only included similar studies published subsequent to the review.

### Item eligibility criteria

Items that were potentially relevant to the risk of bias in NMAs were assessed against the eligibility criteria outlined below. Items related to reporting quality were retained because they potentially could be translated into a risk of bias item.

We included items related to bias, methodological quality or reporting and excluded items that were equally applicable to all systematic reviews as they are covered by other instruments.

#### Exclusion criteria

I. Items that are equally applicable to all systematic reviews as they are covered by other tools (e.g. ROBIS [10], AMSTAR 2 [17]).
II. A tool to assess the risk of bias due to missing evidence in an NMA (i.e. selective outcome reporting and publication bias) has been recently published [35], and we have therefore not included any items related to missing data in an NMA.

Where we included method studies related to NMA biases (e.g. Bujkiewicz 2019 [36]) and studies assessing the quality of NMAs (e.g. Dotson 2019 [37]), we extracted the sentence and surrounding text outlining the method and reworded the text into a concept.

### Search methods for studies

An experienced information specialist executed literature searches in July 2020 in the following electronic databases: MEDLINE (Ovid), Cochrane Library and difficult-to-locate/unpublished (i.e. grey) literature: EQUATOR Network, Dissertation Abstracts, websites (Cochrane, The Canadian Agency for Drugs and Technologies in Health [CADTH], National Institute for Health and Care Excellence [NICE], Pharmaceutical Benefits Advisory Committee, Guidelines International Network, ISPOR and International Network of Agencies for Health Technology Assessment) as well as methods collections (i.e. Cochrane Methodology Register, AHRQ Effective Health Care Program). One expert in search validation designed the search, a second expert revised the search and two librarians independently reviewed the search (Additional file 1).

We scanned the reference lists of included studies. We also asked members of the steering group to identify studies missed by our search. We contacted authors of abstracts or posters to retrieve the full study or when data were missing.

To identify in-house journal editorial standards for NMAs, we created an email list of editors-in-chief of journals publishing NMAs, using the reference list of a bibliometric study of NMAs [38]. We located the journal website using the Google search engine and then located the emails of the editors-in-chief. If they indicated they used an in-house editorial standard for NMAs, then we added these standards to our list of potentially eligible papers.

### Selection of studies

The eligibility criteria were piloted by two reviewers independently on a sample of studies retrieved from the search to ensure consistent application. Two reviewers independently reviewed the title, abstracts, and full text for their potential inclusion against the eligibility criteria. Any disagreement was resolved by discussion with a third reviewer. In instances where there was limited or incomplete information regarding a paper’s eligibility (e.g. when only an abstract was available), the original study authors were contacted to request the full text or further details. Google Translate was used when the authors of the current paper were not fluent in the language of interest.

### Selection of items

Extracted items were reviewed against our eligibility criteria by the steering committee using a consensus-based decision structure. The steering committee decided on their inclusion through an online Zoom™ polling process. The polling options were to include, amend or exclude the item as it was a general systematic review item, or not related to NMA bias.

### Data extraction of studies

From the included studies, we extracted the following data: first author and publication year, standard instrument nomenclature (i.e. tool, scale, checklist and definitions), whether the instrument was designed to assess specific topic areas, number of items, domains within the instrument, whether the instrument focuses on reporting or methodological quality (or focuses on other concepts such as precision of the treatment effect estimates), how domains and items within the instrument are rated (if applicable), methods used to develop the instrument (e.g. review of items, Delphi study, expert consensus meeting) and the availability of guidance as a separate document or included within the original publication.

### Data extraction of items

From the included studies, items potentially relevant to NMAs were extracted verbatim. Two seminal instruments were extracted first because (a) they have the most comprehensive list of items and (b) they were rigorously developed (e.g. used a Delphi process, tested reliability): ISPOR [15] and PRISMA NMA [14] checklists.

PRISMA NMA and ISPOR provided a taxonomy of items, onto which we mapped other similar items (original taxonomy can be found at https://osf.io/f2b5j/). We first (i) extracted items from the ISPOR checklist, (ii) grouped similar PRISMA NMA items next to the ISPOR item and finally (iii) added items not present in ISPOR next to those in the same domain (e.g. eligibility criteria domain). This process made it easier to identify duplicate items, which could be later combined.

Once the items from PRISMA NMA [14] and ISPOR [15] were extracted, a new source was reviewed one at a time based on the year of publication (newest first) [28]. It is hypothesised that old instruments would contain outdated methods and are not as comprehensive.

Once all items were extracted, the following steps were used to group items:

III. Split items so that each item only covers a single concept
IV. Combine duplicate items
V. Group items by similar concept
VI. Categorize items as being related to biases specific to NMAs
VII. Reword into concepts

Two reviewers independently extracted data and discussed discrepancies until a consensus was reached. Data were extracted using Microsoft Excel.

### Organising and categorising items

Several rounds of modification were required until a list of items was finalised and categorised into domains. The steering committee reworded the items, typically structured as questions, into concepts (i.e. general notions) to avoid undue focus on the wording of the item and to make sure these were not confused with a list of items that would be included in the final tool.

### Deviations from the protocol

A deviation from our protocol [31] was that one author (CL) extracted data for the columns “Methods to develop the document” for Tables 3, 4 and 5, and “Research Institute” for Tables 2, 4 and 5, when we had planned for two independent authors to extract all data.

## Results

### Search results

**Fig. 1:**
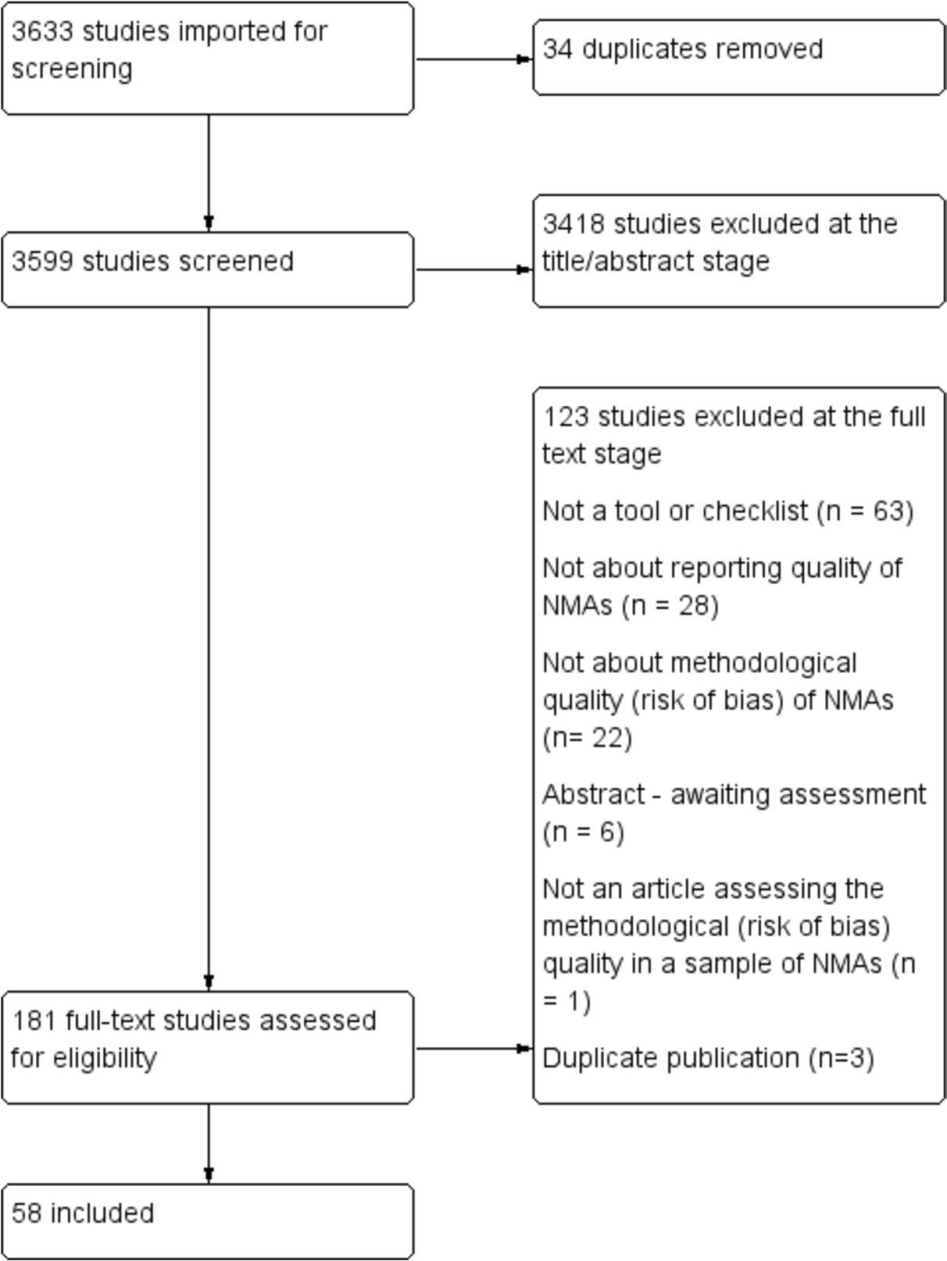
Flowchart of the study selection

The search yielded 3599 citations, 3418 of which were excluded at the title/abstract phase. A total of 181 were assessed in full text and of these, and 58 studies were included (Fig. 1). Three CINeMA studies were similar but reported slightly different results: Nikolakopoulou [23], Papakonstantinou [39] and Salanti [40]. Three articles were therefore grouped together in Table 1.

We identified a review by Laws et al. in 2019 [41] that contained guidance documents for conducting an NMA from countries throughout the world. We therefore did not search for guidance documents published before the last search date of this review. Four other reports were comprehensive methods reviews aggregating previous items related to NMAs [42–45].

### Journal editors’ in-house reporting standards

We located the emails of 206 editors-in-chief of journals publishing NMAs, and of these, 198 emails were successfully delivered. We received 40 responses (40/198), representing a 20% response rate. No respondents reported that they had an in-house editorial standard for NMAs.

### Characteristics of included studies

Of the 58 included studies, 12 were tools, checklists or journal standards; 13 were guidance documents for NMAs; 27 were studies related to bias or NMA methods; and 6 were papers assessing the quality of NMAs.

#### Tools, checklists or standards for NMAs

Two instruments focused solely on the risk of reporting biases, one focused on assessing the validity of NMAs, one focused on assessing certainty in the NMA results, two focused on methodological quality and the remainder mixed all these concepts into one instrument (Table 2). Of the instruments relating to all types of quality or bias, four reported and used rigorous methods in their development (Hutton [14], Jansen [15], Ortega [46], and Page [25]).

**Table 2:** Characteristics of tools, checklists or journal standards (*n* = 12)

First author, year of publication	# items	Type of instrument	Name	Type of assessment	Objective(s)	Research institute	Designed for a specific topic area	Domains within the tool	Rating of items and/or domains	Methods used to develop the tool	Guidance document
Ades 2012 [47]	42	Checklist	NICE DSU checklist	Reporting and methodological quality	Framework for determining whether a convincing argument has been made based on data presented	NICE	Standard meta-analysis, indirect comparisons and NMA	Definition of the decision problem, methods of analysis and presentation of results, issues specific to network synthesis, embedding the synthesis in a probabilistic cost-effectiveness analysis	3 domains: Definition of the decision problem, methods of analysis and presentation of results, issues specific to network synthesis, Embedding the synthesis in a probabilistic cost-effectiveness model	NR	No
Al Khalifah 2018 [48]	11	Checklist	Guide for appraising NMA evidence	Reporting and methodological quality	Users’ guide for pediatricians considering the application of the results of NMA	McMaster University	NMA	Credibility of NMA methods, certainty of NMA evidence, were results consistent across studies, how trustworthy are the indirect comparisons, applicability	NA	NR	No
Dias 2018 [49]	14	Checklist		Validity of NMAs	Introduce and discuss validity of NMAs	University of Bristol	NMA	Question formulation, trial inclusion/ exclusion and network connectivity; heterogeneity and bias management; reporting	NA	NR	No
Hutton 2015 [14]	32	Checklist	PRISMA NMA	Reporting	Present the NMA PRISMA extension and provide examples of good reporting	Ottawa Hospital Research Institute	Systematic reviews with NMA	Title, abstract, introduction, methods, results, discussion, funding	NA	Overview of reviews, Delphi, expert opinion	No
Jansen 2011 [50]	21	Checklist	Simplified checklist to assist decision makers in evaluating a reported NMA	Reporting and methodological quality	Provide guidance on the interpretation of indirect treatment comparisons and NMA to assist policymakers and health-care professionals in using its findings for decision making	ISPOR	NMA	Introduction, methods, results, discussion	NA	NR	No
Jansen 2014 [15]	26	Questionnaire	ISPOR	Reporting and methodological quality	Help decision makers assess the relevance and credibility of indirect treatment comparisons and NMA	ISPOR	NMA	Evidence base, analysis, reporting quality and transparency, interpretation, conflict of interest	3 levels: yes, no, cannot answer	Expert opinion, literature search, pilot testing	No
Kiefer 2015 [51]	9	Checklist	Checklist for evaluation of indirect comparisons and network meta-analyses	Reporting and methodological quality	Describe the underlying assumptions and methods used in indirect comparisons and NMA and explain what evaluation of such publications should include	Institute for Quality and Efficiency in Health Care (IQWiG)	NMA	Methods, statistical analysis, reporting, limitations	NA	NA	No
Nikolakopoulou 2020 [23], Papakonstantinou 2020 [39], Salanti 2014 [40]	6	Framework	CINeMA	Confidence in results from NMA	Evaluate confidence in the results from network meta-analyses	Cochrane and the Campbell collaboration	NMA	Within-study bias, reporting bias, indirectness, imprecision, heterogeneity and incoherence	3 levels: no concerns, some concerns or major concerns (within); 4 levels: high, moderate, low, very low (summary)	Developed based on three previous studies, and participant feedback	Yes
Ortega 2014 [46]	20	Checklist	Checklist for critical appraisal of indirect comparisons	Reporting and methodological quality	Critical appraisal of indirect comparisons of drugs, considering clinical, methodological/statistical and quality aspects, applied in drug evaluation in the decision-making	Clinica Universidad de Navarra	Indirect comparisons	Quality, clinical aspects, methodology/statistics	3 levels: high, acceptable, low	Review of literature, group consensus, expert guidance	No
Page 2020 [25]	27	Checklist	PRISMA 2020 Statement	Reporting	Describe and justify changes made to the guideline	Monash University	Systematic reviews	Title, abstract, introduction, methods, results, discussion, other information	NA	Review, survey, expert meeting	Yes

*CINeMA* Confidence in Network Meta-Analysis, *DSU* Decision Support Unit, *GRADE-NMA* Grading of Recommendations Assessment, Development and Evaluation for Network Meta-Analysis, *ISPOR* International Society for Pharmacoeconomics and Outcomes Research, *NA* not applicable, *NICE* National Institute for Health and Care Excellence, *NR* not reported, *OQAQ* Overview Quality Assessment Questionnaire, *PRISMA* Preferred Reporting Items for Systematic Reviews and Meta-Analyses

Nearly all of the included tools (*n* = 10/12) were domain-based, where users judge the risk of bias or methodological quality within specific domains (Table 2). All NMA tools were designed for generic rather than specific use (e.g. a tool designed only for meta-analyses of diagnostic accuracy studies). Six tools described methods to develop the tool, or linked to supplementary data containing this information. Five of the tools included guidance documents.

#### Guidance documents for NMAs

We identified 13 guidance documents for the conduct and reporting of NMAs (Table 3). None of the guidance reports was targeted at specific types of NMAs. One study by Laws in 2019 [41] was a comprehensive systematic review of all guidance for NMAs worldwide, and none of which was targeted at specific types of NMAs. In the Laws systematic review [41], guidelines from 41 countries were examined, yielding guideline documents from 14 countries that were related to the conduct of an NMA. Laws [41] broadly categorized the criteria for conducting NMA from these guidelines as (a) assessments and analyses to test assumptions required for an NMA, (b) presentation and reporting of results and (c) justification for modeling choices.

**Table 3:** Characteristics of guidance documents (*n* = 13)

First author, year of publication	Title	Objective	Research institute	Country of the first author	Methods used to develop the guidance
Brignardello-Petersen 2018 [22]	Advances in the GRADE approach to rate the certainty in estimates from a network meta-analysis	Present recent advances to grade the certainty of the evidence	GRADE Working Group	Canada	NR
CADTH 2015 [52]	Guidance document on reporting indirect comparisons	Provide guidance on reporting indirect comparisons	CADTH	Canada	NR
Chaimani 2019 [53]	Undertaking network meta-analyses	Introduce/provide an overview of concepts, assumptions and methods of NMAs	Cochrane	UK	NR
Chaimani 2017a [54]	Additional considerations are required when preparing a protocol for a systematic review with multiple interventions	Highlight aspects of a standard systematic review protocol that may need modification when multiple interventions are to be compared	University of Ioannina School of Medicine	Greece	NR
Chaimani 2017b [55]	Common pitfalls and mistakes in the set-up, analysis and interpretation of results in network meta-analysis: what clinicians should look for in a published article	Provide a practical framework to assess the methodological robustness and reliability of results from network meta-analysis	University of Ioannina School of Medicine	Greece	NR
Coleman 2012 [56]	Use of mixed treatment comparisons in systematic reviews	Summarise available guidance for meta-analytic methods, identify analyses using these methods and summarize their characteristics, and identify rationale for selection/implementation/reporting of methods from investigators	Agency for Healthcare Research and Quality (AHRQ)	United States	Review guidance documents and MTC literature, expert opinion
Cope 2014 [57]	A process for assessing the feasibility of a network meta-analysis: a case study of everolimus in combination with hormonal therapy versus chemotherapy for advanced breast cancer	Outline a general process for assessing the feasibility of performing a valid NMA of RCT	Mapi	Canada	NR
Dwan 2020 [58]	Editorial decisions in reviews with network meta-analysis	Present editorial considerations in reviews with NMA	Cochrane	UK	NR
Foote 2015 [59]	Network Meta-analysis: Users' Guide for Surgeons: Part I—Credibility	Show the application of evaluation criteria for determining the credibility of a NMA through an example pertinent to clinical orthopaedics	McMaster University	Canada	NR
Haute Autorité de Santé 2009 [60]	Indirect comparisons Methods and validity	Introduce and discuss indirect comparison methods	Haute Autorité de Santé	France	Review of literature, expert peer review
Hummela 2017 [61]	Work Package 4: Methodological guidance, recommendations and illustrative case studies for (network) meta-analysis and modelling to predict real-world effectiveness using individual participant and/or aggregate data	Summarise state-of-the-art methods in NMA, IPD meta-analysis and mathematical modelling to predict drug effectiveness based on RCT data and related software, and discuss their advantages and limitations	University of Bern	Switzerland	Review of literature
Laws 2019 [41]	A Comparison of National Guidelines for Network Meta-Analysis	Create a superset of requirements collated from available national guidelines for the conduct of NMAs	Amaris	UK	Review of literature
Welton 2020 [45]	Sources and synthesis of evidence; Update to evidence synthesis methods (CHTE2020)	Review existing and emerging methods for synthesising evidence on clinical effectiveness for decision-making in Health Technology Appraisals (HTA), including NMA	NICE Decision Support Unit	UK	NR

*CADTH* Canadian Agency For Drugs And Technologies In Health, *NA* not applicable, *NICE-DSU* National Institute for Health and Care Excellence Decision Support Unit checklist, *NR* not reported, *MTC* multiple treatment comparisons

#### Studies assessing the methodological or reporting quality of NMAs

Of the six papers assessing the quality of NMAs, one assessed reporting quality using PRISMA NMA [62] (Table 4). Three assessments used the National Institute for Health and Care (NICE) Guide to the Methods of Technology Appraisal, the NICE Excellence Decision Support Unit checklist (NICE-DSU) alone [63], or the latter in combination with the ISPOR checklist [64, 65]. The remaining two studies did not report basing their assessment on any instrument; Donegan [66] assessed both methodological quality and reporting quality but did not base their assessment on an established instrument, and Dotson [37] evaluated if NMAs displayed evidence of a confounding bias that varies with time.

**Table 4:** Characteristics of studies assessing quality of NMAs (*n* = 6)

First author, year of publication	Title	Objective	Research institute	Country of the first author	Name of the tools used	Type of assessment	Assessed effectiveness, efficacy and/or safety	Clinical focus	Methods used to develop the study	Authors’ conclusions
Bafeta 2014 [64]	Reporting of results from network meta-analyses: methodological systematic review	Examine how network meta-analysis results are reported	Hôpital Hôtel-Dieu	France	NICE DSU and ISPOR	Reporting	Effectiveness, efficacy, and safety	NA	Meta-research study of NMAs	NMAs are heterogeneously reported. Development of reporting guidelines for critically appraising reports of NMAs is timely
Donegan 2010 [66]	Indirect comparisons: a review of reporting and methodological quality	Report a systematic review of the reporting and methodological quality of published indirect comparisons using specifically devised quality assessment criteria	University of Liverpool	UK	Not reported	Methodological quality	Effectiveness	NA	Meta-research study of NMAs	The underlying assumptions are not routinely explored or reported when undertaking indirect comparisons. We recommend that the quality should be improved by assessing assumptions and reporting the assessment methods applied
Dotson 2019 [37]	Rising placebo response rates threaten the validity of antipsychotic meta-analyses	Evaluate if NMAs display evidence of a confounding bias that varies with time	University of Liverpool	USA	Not reported	Risk of bias	Efficacy	Anti-psychotics/anti-depressants	Meta-research study of NMAs	Rankings of antipsychotics, but not antidepressants, show evidence of a confounding temporal bias. Poorly compensated placebo inflation is one potential explanation for this finding
Fleetwood 2016 [63]	A Review of the Use of Network Meta-Analysis In Nice Single Technology Appraisals	Evaluate the use of NMA within Single Technology Appraisals (STAs) with respect to the NICE guidance	Quantics Consulting Ltd	UK	NICE DSU	Reporting and methodological quality	Effectiveness	NA	Meta-research study of NMAs	Although STAs often include NMAs, these do not always entirely conform to the NICE guidelines. Manufacturers should present all of the information recommended by the NICE guidelines
Thieffry 2020 [65]	Understanding the challenge of comparative effectiveness research in focal epilepsy: A review of network meta-analyses and real-world evidence on antiepileptic drugs (AED)	Building on previous assessments of NMAs of AEDs as adjunctive treatment of focal seizures, we review the meth-odological quality and robustness of recent NMAs	UCB Pharma	Belgium	NICE DSU, ISPOR	Methodological quality	Efficacy, safety	Focal seizures	Meta-research study of NMAs	Current NMAs provide only nominal comparative evidence for AED treatments and should be used with caution for decision-making due to their methodological limitations
			Federal University of Parana							
Tonin 2018 [62]	Mapping the characteristics of network meta-analyses on drug therapy: A systematic review	Our aim was to map the characteristics of all the NMAs published, including drug therapy comparisons	Federal University of Parana	Brazil	PRISMA NMA	Reporting and methodological quality	Not reported	Drug therapy comparisons	Meta-research study of NMAs	The map can gather NMA evidence, but it also identified some weaknesses, especially in the report, which limits its transparency and reproducibility

*AED* antiepileptic drugs, *ISPOR* International Society for Pharmacoeconomics and Outcomes Research, *NA* not applicable, *NICE* National Institute for Health and Care Excellence, *NR* not reported, *STAs* Single Technology Appraisal

#### Method and bias studies on NMAs

Of the 27 papers on methods for NMAs, 11 were from the UK, 8 were from Canada and the USA each, 2 were from Germany, Switzerland and Greece each, and one each was from Ireland and Portugal. The majority of methods studies were not aimed at a specific type of NMA, nor a specific medical field (*n* = 18/27). Of the five studies that focused on a specific type of NMA, two were aimed at disconnected networks, and one each of adaptive trial designs, random inconsistency effects and Bayesian models (Table 5). The remaining four were aimed at specific medical fields, namely depression, hypertension, social anxiety, any drug therapy and inflammatory arthritis.

**Table 5:** Table of characteristics of methods studies related to NMA biases (*n* = 27)

First author, year of publication	Title	Objective	Research Institute	Country of the first author	Specific topic area	Methods used
Cameron 2017 [67]	Importance of Considering Differences in Study Design in Network Meta-analysis: An Application Using Anti-Tumor Necrosis Factor Drugs for Ulcerative Colitis	Present approaches to adjust findings derived from adaptive study designs to make them more comparable with findings derived from conventional parallel design RCTs	Cornerstone Research Group	Canada	Adaptive trial design RCT	Case study
Cameron 2018 [68]	Importance of assessing and adjusting for cross-study heterogeneity in network meta-analysis: a case study of psoriasis	Present adjustment for cross-study heterogeneity when conducting NMAs using a case study of biologic therapies for moderate-to-severe plaque psoriasis	Cornerstone Research Group	Canada	No	Case study
Davies 2021 [69]	Degree irregularity and rank probability bias in network meta-analysis	Study how the structure of the network affects the probability that each treatment is ranked first, second and so on	University of Manchester	UK	No	Case study
Donegan 2013 [42]	Assessing key assumptions of network meta-analysis: a review of methods	Review and illustrate methods to assess homogeneity and consistency	University of Liverpool	UK	No	Review and case study
Efthimiou 2020 [70]	The dark side of the force: Multiplicity issues in network meta-analysis and how to address them	Present problems with the usual standard models for NMA in estimating multiple relative treatment effects, and suggest alternative modelling methods	University of Bern	Switzerland	No	Review of the literature
Efthimiou 2016 [43]	GetReal in network meta-analysis: a review of the methodology	Present methods for NMA and discuss conceptual and statistical ways of evaluating the underlying assumptions of the model while providing guidance for researchers	University of Ioannina School of Medicine	Greece	No	Theoretical arguments and simulations
Efthimiou 2017 [71]	Multivariate extension of meta-analysis	Objective 1. Provide a comprehensive account of the currently available methods. Objective 2. Advance the statistical methodology for jointly analyzing multiple correlated outcomes in NMAs	University of Ioannina	Greece	No	Review and case study
Goring 2016 [72]	Disconnected by design: analytic approach in treatment networks having no common comparator	Describe disconnected networks, present alternative Bayesian models and provide a framework to guide the choice between modeling approaches	ICON plc	Ireland	Disconnected networks	Theoretical arguments and simulations
Jackson 2017 [73]	Paule-Mandel estimators for network meta-analysis with random inconsistency effects	Discuss and simulate Paule-Mandel estimators for NMA with random inconsistency effects	MRC Biostatistics Unit	UK	Random inconsistency effects	Theoretical arguments and simulations
Jansen 2011 [12]	Is network meta-analysis as valid as standard pairwise meta-analysis? It all depends on the distribution of effect modifiers	Compare pairwise meta-analysis with network meta-analysis with a specific focus on the primary role of effect modifiers as the common cause of heterogeneity and bias	Mapi Group	USA	No	Theoretical arguments
Kibret 2014 [74]	Bias in the identification of the best treatment in a Bayesian network meta-analysis for binary outcome: a simulation study	Investigate how rank probabilities obtained from a Bayesian NMA are affected by characteristics of the network, including network configuration, number of studies per comparison, individual study sample sizes, and effect sizes	McMaster University	Canada	Bayesian framework	Theoretical arguments and simulation
Krahn 2013 [75]	A graphical tool for locating inconsistency in network meta-analyses	Provide a tool, the net heat plot, to render transparent which direct comparisons drive each network estimate and to display hot spots of inconsistency	Johannes Gutenberg University	Germany	No	Theoretical arguments and case studies
Lin 2016 [76]	Sensitivity to Excluding Treatments in Network Meta-analysis	Examine the sensitivity to treatment exclusion of an alternative approach to network meta-analysis, namely the arm-based approach	University of Minnesota	USA	Arm-based approach	Theoretical arguments and case study
Linde 2016 [77]	Questionable assumptions hampered interpretation of a network meta-analysis of primary care depression treatments	Evaluate the underlying assumptions of a network meta-analysis and highlight challenges and pitfalls of interpretation under consideration of the assumptions	Technische Universitat Munchen	Germany	Effectiveness and acceptability of pharmacologic and psychological treatments for primary care patients with depression	Case study
Marks-Anglin 2020 [78]	A historical review of publication bias	Offer an historical account of seminal contributions, with an emphasis on the parallel development of graph-based and selection model approaches	University of Pennsylvania	USA	No	Review and theoretical arguments
Naci 2014 [79]	Industry sponsorship bias in research findings: a network meta-analysis of LDL cholesterol reduction in randomised trials of statins	Explore the risk of industry sponsorship bias	London School of Economics and Political Science	UK	Placebo-controlled and active comparator trials of statins	Case study
Owen 2020 [80]	Multivariate network meta-analysis incorporating class effects	Extend the trivariate NMA model to incorporate the exchangeability between class treatment effects, and a missing data framework to estimate uncertainty in trials that did not report measures of variability	University of Leicester	UK	No	Theoretical arguments and case studies
Papakonstantinou 2020 [81]	In network meta-analysis most of the information comes from indirect evidence: empirical study	Examine the relative contribution of network paths of different lengths to estimates of treatment effects	University of Bern	Switzerland	No	Meta-research study of NMAs
Phillippo 2019 [24]	Threshold Analysis as an Alternative to GRADE for Assessing Confidence in Guideline Recommendations Based on Network Meta-Analyses	Outline threshold analysis as an alternative approach, demonstrating the method with two examples of clinical guidelines	NICE Guidelines Technical Support Unit	UK	Social anxiety and depression guidelines	Theoretical arguments and case studies
Salanti 2009 [82]	A case study of multiple-treatments meta-analysis demonstrates that covariates should be considered	Illustrate the simultaneous analysis of a network of trials, using as a case study	MRC Biostatistics Unit	UK	Topical fluoride treatments	Case study
Shi 2018 [83]	Node-making processes in network meta-analysis of nonpharmacological interventions should be well planned and reported	Describe four ways to create a network of nodes based on NMA objectives	University of Manchester	UK	Nonpharmacological interventions	Theoretical argument
Song 2003 [84]	Validity of indirect comparison for estimating efficacy of competing interventions: empirical evidence from published meta-analyses	Determine the validity of adjusted indirect comparisons by using data from published meta-analyses of randomised trials	University of Birmingham	UK	No	Meta-research study of NMAs
Stevens 2018 [44]	A review of methods for comparing treatments evaluated in studies that form disconnected networks of evidence	Review and discuss methods for comparing treatments evaluated in studies that form disconnected networks of evidence	University of Sheffield	UK	Disconnected networks	Review of methods and applications
Swallow 2020 [85]	Causal inference and adjustment for reference-arm risk in indirect treatment comparison meta-analysis	Outline methods to reduce biases associated with ITC/NMA and apply them to three real-world examples	Analysis Group Inc	USA	No	Theoretical arguments and case studies
Tan 2013 [86]	Presentational approaches used in the UK for reporting evidence synthesis using indirect and mixed treatment comparisons	Provide recommendations to improve indirect comparison/mixed treatment comparison reporting and identify research priorities for improved presentation	University of Leicester	UK	No	Review of guidance
Thorlund 2013 [87]	Why the findings of published multiple treatment comparison meta-analyses of biologic treatments for rheumatoid arthritis are different: an overview of recurrent methodological shortcomings	Evaluate the quality of published MTCs and to identify methodological issues that can explain the discrepancies in the findings of these MTCs	McMaster University	Canada	Biologic disease-modifying antirheumatic drugs for rheumatoid arthritis	Meta-research study of NMAs
Tonin 2019 [88]	Description of network meta-analysis geometry: A metrics design study	We aimed to propose metrics adapted from graph theory and social network-analysis literature to numerically describe NMA geometry	Federal University of Parana	Portugal	Any drug therapy intervention	Meta-research study of NMAs

*MTC* multiple treatment comparisons, *NA* not applicable, *NR* not reported

### Retained concepts

A total of 99 items were extracted verbatim from the 58 studies (dataset at https://osf.io/f2b5j/), and after item screening against the eligibility criteria, we included 22 that were reworded into concepts (Additional file 3).

The concepts in Additional file 3 were categorised into the following domains: 3 concepts in network characteristics, 4 concepts in effect modifiers, 13 concepts in statistical synthesis and 2 concepts in interpretation of the findings and conclusions. Concepts related to joint randomisability, inappropriate exclusion of interventions, specification of nodes, network geometry, effect modifiers, appropriate handling of multi-arm studies, heterogeneity, consistency, choice of priors, sensitivity analyses, robustness of the results and trustworthiness of the conclusions were considered. These concepts should not be used to assess bias in NMAs as they are preliminary thoughts which will be altered and refined into items based on expert feedback [89].

## Discussion

Using a systematic search of the literature, we identified 58 studies presenting items or concepts related to quality or bias in NMAs. When we surveyed editors-in-chief of journal publishing NMAs, we found that none reported using in-house editorial standards for NMAs. These studies yielded 99 items of which the majority related to general systematic review biases and quality, which are covered in tools such as AMSTAR 2 [17] and ROBIS [90] and were therefore excluded. Twenty-two concepts related to biases specific to NMAs were retained. Concepts related to joint randomisability, effect modifiers, specification of nodes, inconsistency, robustness of the results, and trustworthiness of the conclusions, and others were considered. The list of concepts in Additional file 3 is not intended to be used as an instrument. While waiting for our tool to be finalised and published, stakeholders should use a combination of methods and topical expertise to anticipate the most important sources of bias, assess risk of bias and interpret the effect of potential sources of bias on NMA estimates of effect and authors’ conclusions.

### Strengths and limitations

A major strength of our research was that we conducted it in accordance with a systematic review protocol [31]. Two other studies, Sanderson [30] and Page [28], developed lists of quality items systematically. We followed their methods which involved building a bank of items through a systematic review of the relevant literature. Other strengths included using a systematic search strategy developed by an information specialist and inclusion of grey literature in any language, using intuitive domains to organise items related to bias and using a consensus-based decision structure to select, reframe and refine items.

One limitation of our study is the challenge in retrieving methods studies as methods collections are not regularly updated (for example, the Cochrane Methodology Register has not been updated since July 2012 [91] and the Scientific Resource Center Methods library’s most recent article is from 2013). Since the submission of this manuscript, two new websites for methods guidance have emerged: LIGHTS (https://lights.science/) for methods guidance and LATITUDES (www.latitudes-network.org) which features validity assessment tools. However, we do not expect any missing relevant methods studies or tools to supply additional novel concepts.

An additional limitation is that potentially relevant studies may have been published since our last search (July 2020), and our search may not have retrieved all relevant studies. However, the 22 included concepts reflect all aspects of NMA bias considered by previous methodological tools and their expert authors, and it is therefore unlikely that important concepts are missing.

### Impact of the development of a new risk of bias tool for NMAs

We believe our proposed tool to assess the risks of bias in NMA is needed for several reasons. Other tools and checklists for NMAs have been published; however, few of these were developed based on systematic and rigorous methodology (i.e. Moher [26] and Whiting [27]), and none is current and comprehensive (see Table 1). The PRISMA-NMA (Hutton [14]) and the NICE-DSU checklist (Ades [47]) were designed to assess reporting quality (i.e. how well a study is described in publication). The ISPOR checklist (Jansen [15]) was designed to assess reporting, validity and applicability. Finally, the checklist for critical appraisal of indirect comparisons (Ortega [46]) was designed to assess methodological quality. These tools (published between 2012 and 2014) are now outdated and fail to incorporate advances in biases, methodological and statistical approaches to NMA evidence synthesis. Our proposed tool will be current and aims to incorporate these new advances.

### Future research

This study represents the first stage in the development of a new risk of bias tool for NMAs. This systematic review of items identified 22 concepts which were entered into a Delphi survey to solicit expert opinion [89]. The steering committee used expert feedback to choose and refine the concepts. We also considered feedback from a stakeholder survey on the structure, conceptual decisions and concepts in the proposed tool [89]. The concepts were then worded into items, and an elaboration and explanation document was written. The protocol tool is currently undergoing pilot testing, and those interested in piloting, or using the tool in the future, can contact the first author (CL). The steering committee intended the RoB NMA tool to be used in combination with ROBIS [10] (which we recommend as it was designed to assess biases specifically) or other similar tools (e.g. AMSTAR 2 [17]) to assess the quality of systematic reviews. Further research will involve reliability and validity testing.

## Conclusions

Twenty-two concepts were included, which will inform the development of a new tool to assess the risk of bias in NMAs. Concepts related to joint randomisability, effect modifiers, specification of nodes, inconsistency, robustness of the results, and trustworthiness of the conclusions and others were considered. The list of concepts is not intended to be used as an instrument to assess biases in NMAs, but to inform the development of items to be included in our tool.

### Supplementary Information

**Additional file 1:** **Appendix 1.** Steering committee. **Appendix 2.** Search strategies (July 2020). **Appendix 3.** Retained concepts.

## Data Availability

The datasets used and/or analysed during the current study are available freely at https://osf.io/f2b5j/.

## References

[1] Créquit P (2016). Wasted research when systematic reviews fail to provide a complete and up-to-date evidence synthesis: the example of lung cancer. BMC Med.

[2] Gotzsche PC. Why we need a broad perspective on meta-analysis. It may be crucially important for patients. BMJ. 2000;321(7261):585–6. 10.1136/bmj.321.7261.585. 10.1136/bmj.321.7261.585 PMC1118486 10977820

[3] Ioannidis JP (2009). Integration of evidence from multiple meta-analyses: a primer on umbrella reviews, treatment networks and multiple treatments meta-analyses. CMAJ.

[4] Leucht S (2016). Network meta-analyses should be the highest level of evidence in treatment guidelines. Eur Arch Psychiatry Clin Neurosci.

[5] Li T (2016). Comparative effectiveness of first-line medications for primary open-angle glaucoma: a systematic review and network meta-analysis. Ophthalmology.

[6] Nikolakopoulou A (2018). Living network meta-analysis compared with pairwise meta-analysis in comparative effectiveness research: empirical study. BMJ.

[7] Naudet F, Schuit E, Ioannidis J (2017). Overlapping network meta-analyses on the same topic: survey of published studies. Int J Epidemiol.

[8] Patel CJ, Burford B, Ioannidis JP (2015). Assessment of vibration of effects due to model specification can demonstrate the instability of observational associations. J Clin Epidemiol.

[9] Chandler, J., et al., Methodological standards for the conduct of new Cochrane Intervention Reviews. Sl: Cochrane Collaboration, 2013.

[10] Whiting P (2016). ROBIS: a new tool to assess risk of bias in systematic reviews was developed. J Clin Epidemiol.

[11] Greco T (2015). The attractiveness of network meta-analysis: a comprehensive systematic and narrative review. Heart Lung Vessels.

[12] Jansen JP, Naci H (2013). Is network meta-analysis as valid as standard pairwise meta-analysis? It all depends on the distribution of effect modifiers. BMC Med.

[13] Li T (2011). Network meta-analysis-highly attractive but more methodological research is needed. BMC Med.

[14] Hutton B (2015). The PRISMA extension statement for reporting of systematic reviews incorporating network meta-analyses of health care interventions: checklist and explanations. Ann Intern Med.

[15] Jansen JP (2014). Indirect treatment comparison/network meta-analysis study questionnaire to assess relevance and credibility to inform health care decision making: an ISPOR-AMCP-NPC good practice task force report. Value Health.

[16] Higgins JP (2011). The Cochrane collaboration’s tool for assessing risk of bias in randomised trials. BMJ.

[17] Shea BJ (2017). AMSTAR 2: a critical appraisal tool for systematic reviews that include randomised or non-randomised studies of healthcare interventions, or both. BMJ..

[18] Shea BJ (2007). Development of AMSTAR: a measurement tool to assess the methodological quality of systematic reviews. BMC Med Res Methodol.

[19] Oxman AD, Guyatt GH (1991). Validation of an index of the quality of review articles. J Clin Epidemiol.

[20] Guyatt G (2011). GRADE guidelines: 1. Introduction—GRADE evidence profiles and summary of findings tables. J Clin Epidemiol.

[21] Puhan MA (2014). A GRADE Working Group approach for rating the quality of treatment effect estimates from network meta-analysis. BMJ.

[22] Brignardello-Petersen R (2018). Advances in the GRADE approach to rate the certainty in estimates from a network meta-analysis. J Clin Epidemiol.

[23] Nikolakopoulou A (2020). CINeMA: an approach for assessing confidence in the results of a network meta-analysis. PLoS Med/ Publ Library Sci.

[24] Phillippo DM (2019). Threshold analysis as an alternative to GRADE for assessing confidence in guideline recommendations based on network meta-analyses. Ann Intern Med.

[25] Page MJ (2021). The PRISMA 2020 statement: an updated guideline for reporting systematic reviews. BMJ.

[26] Moher D (2010). Guidance for developers of health research reporting guidelines. PLoS Med.

[27] Whiting P (2017). A proposed framework for developing quality assessment tools. Syst Rev.

[28] Page MJ (2020). Mapping of reporting guidance for systematic reviews and meta-analyses generated a comprehensive item bank for future reporting guidelines. J Clin Epidemiol.

[29] Song F (2009). Methodological problems in the use of indirect comparisons for evaluating healthcare interventions: survey of published systematic reviews. BMJ.

[30] Sanderson S, Tatt ID, Higgins J (2007). Tools for assessing quality and susceptibility to bias in observational studies in epidemiology: a systematic review and annotated bibliography. Int J Epidemiol.

[31] Lunny C (2021). Methodological review to develop a list of bias items used to assess reviews incorporating network meta-analysis: protocol and rationale. BMJ Open.

[32] Page MJ, McKenzie JE, Higgins JPT (2018). Tools for assessing risk of reporting biases in studies and syntheses of studies: a systematic review. BMJ Open.

[33] Moher D (1995). Assessing the quality of randomized controlled trials: an annotated bibliography of scales and checklists. Control Clin Trials.

[34] Chambers JD, Naci H, Wouters OJ, Pyo J, Gunjal S, Kennedy IR, Hoey MG, Winn A, Neumann PJ. An assessment of the methodological quality of published network meta-analyses: a systematic review. PLoS One. 2015;10(4):e0121715. 10.1371/journal.pone.0121715. Erratum in: PLoS One. 2015;10(7):e0131953. 10.1371/journal.pone.0121715 PMC4414531 25923737

[35] Chiocchia V (2021). ROB-MEN: a tool to assess risk of bias due to missing evidence in network meta-analysis. BMC Med.

[36] Bujkiewicz, S., F. Achana, T. Papanikos, R. D. Riley, and K. R. Abrams. "NICE DSU Technical Support Document 20: multivariate meta-analysis of summary data for combining treatment effects on correlated outcomes and evaluating surrogate endpoints. 2019." Availabe from: https://www.sheffield.ac.uk/nice-dsu/tsds/full-list.

[37] Dotson S (2019). Rising placebo response rates threaten the validity of antipsychotic meta-analyses. Ann Clin Psychiatry.

[38] Ban JK (2018). History and publication trends in the diffusion and early uptake of indirect comparison meta-analytic methods to study drugs: animated coauthorship networks over time. BMJ Open.

[39] Papakonstantinou T (2018). Estimating the contribution of studies in network meta-analysis: paths, flows and streams. F1000Research..

[40] Salanti G. et al. Evaluating the quality of evidence from a network meta-analysis. PloS one. 2014;9(7). 10.1371/journal.pone.0099682 PMC4084629 24992266

[41] Laws A, Tao R, Wang S, Padhiar A, Goring S. A comparison of national guidelines for network meta-analysis. Value in Health. 2019;22(10):1178–86. 10.1016/j.jval.2019.05.013 31563261

[42] Donegan S (2013). Assessing key assumptions of network meta-analysis: a review of methods. Res Synth Methods.

[43] Efthimiou O (2016). GetReal in network meta-analysis: a review of the methodology. Res Synth Methods.

[44] Stevens JW (2018). A review of methods for comparing treatments evaluated in studies that form disconnected networks of evidence. Res Synth Methods.

[45] Welton NJ, Phillippo DM, Owen R, Jones HE, Dias S, Bujkiewicz S, Ades AE, Abrams KR. CHTE2020 sources and synthesis of evidence; update to evidence synthesis methods. Sheffield: National Institute for Health and Care Excellence (NICE) Decision Support Unit (DSU). 2020. http://rees-france.com/wp-content/uploads/2020/12/CHTE-2020_synthesis-of-evidence.pdf.

[46] Ortega A (2014). A checklist for critical appraisal of indirect comparisons. Int J Clin Pract.

[47] Ades, A., et al., NICE DSU Technical Support Document 7: Evidence synthesis of treatment efficacy in decision making: a reviewer’s checklist. 2012, National Institute for Health and Clinical Excellence: https://research-information.bris.ac.uk/en/publications/nice-dsu-technical-support-document-7-evidence-synthesis-of-treatment-efficacy-in-decision-making-a-reviewers-checklist(3831c37d-b492-446f-8882-d94cabf7b95d).html. p. 01. 27905719

[48] Al Khalifah R (2018). Network meta-analysis: users' guide for pediatricians. BMC Pediatr.

[49] Dias S. et al. Chapter 8 Validity of network meta-analyses. In: Network meta-analysis for decision-making. 2018: Wiley.

[50] Jansen JP (2011). Interpreting indirect treatment comparisons and network meta-analysis for health-care decision making: report of the ISPOR task force on indirect treatment comparisons good research practices: part 1. Value in Health.

[51] Kiefer C, Sturtz S, Bender R (2015). Indirect comparisons and network meta-analyses. Deutsches Arzteblatt international.

[52] Richter T, Lee KA (2015). and CADTH Working Group Contributors, Guidance document on reporting indirect comparisons.

[53] Chaimani A. et al. Undertaking network meta-analyses. Cochrane handbook for systematic reviews of interventions. 2019:285–320.

[54] Chaimani A (2017). Additional considerations are required when preparing a protocol for a systematic review with multiple interventions. J Clin Epidemiol.

[55] Chaimani A (2017). Common pitfalls and mistakes in the set-up, analysis and interpretation of results in network meta-analysis: what clinicians should look for in a published article. Evid Based Ment Health.

[56] Coleman CI (2012). AHRQ methods for effective health care, in use of mixed treatment comparisons in systematic reviews.

[57] Cope S (2014). A process for assessing the feasibility of a network meta-analysis: a case study of everolimus in combination with hormonal therapy versus chemotherapy for advanced breast cancer. BMC Med.

[58] Dwan, K., Bickerdike, L., Livingstone, N., Editorial decisions in reviews with network meta-analysis. https://training.cochrane.org/resource/editorial-considerations-reviews-network-meta-analysis. 2020, Cochrane Editorial and Methods Department.

[59] Foote CJ (2015). Network meta-analysis: users' guide for surgeons: part I - credibility. Clin Orthop Relat Res.

[60] Haute Autorité de Santé, Summary Report. Indirect comparisons, methods and validity. 2009.

[61] Hummela N. et al., Work Package 4 Methodological guidance, recommendations and illustrative case studies for (network) meta-analysis and modelling to predict real-world effectiveness using. 2017.

[62] Tonin FS (2018). Mapping the characteristics of network meta-analyses on drug therapy: a systematic review. PLoS ONE [Electronic Resource].

[63] Fleetwood K (2016). A review of the use of network meta-analysis in NICE single technology appraisals. Value Health.

[64] Bafeta A (2014). Reporting of results from network meta-analyses: methodological systematic review. BMJ.

[65] Thieffry S (2020). Understanding the challenge of comparative effectiveness research in focal epilepsy: a review of network meta-analyses and real-world evidence on antiepileptic drugs. Epilepsia.

[66] Donegan S (2010). Indirect comparisons: a review of reporting and methodological quality. PLoS ONE.

[67] Cameron C (2017). The importance of considering differences in study design in network meta-analysis: an application using anti-tumor necrosis factor drugs for ulcerative colitis. Med Decis Making.

[68] Cameron C (2018). Importance of assessing and adjusting for cross-study heterogeneity in network meta-analysis: a case study of psoriasis. J Compar Effect Res.

[69] Davies AL, Galla T (2021). Degree irregularity and rank probability bias in network meta-analysis. Res Synth Methods.

[70] Efthimiou O, White IR (2020). The dark side of the force: multiplicity issues in network meta-analysis and how to address them. Res Synth Methods.

[71] Efthimiou O. Multivariate extension of meta-analysis. 2017, Πανεπιστήμιο Ιωαννίνων. Σχολή Επιστημών Υγείας. Τμήμα Ιατρικής. Τομέας ….

[72] Goring S (2016). Disconnected by design: analytic approach in treatment networks having no common comparator. Res Synth Methods.

[73] Jackson D (2017). Paule-Mandel estimators for network meta-analysis with random inconsistency effects. Res Synth Methods.

[74] Kibret T, Richer D, Beyene J (2014). Bias in identification of the best treatment in a Bayesian network meta-analysis for binary outcome: a simulation study. Clin Epidemiol.

[75] Krahn U, Binder H, König J (2013). A graphical tool for locating inconsistency in network meta-analyses. BMC Med Res Methodol.

[76] Lin L, Chu H, Hodges JS (2016). Sensitivity to excluding treatments in network meta-analysis. Epidemiology (Cambridge, Mass).

[77] Linde K (2016). Questionable assumptions hampered interpretation of a network meta-analysis of primary care depression treatments. J Clin Epidemiol.

[78] Marks-Anglin A, Chen Y (2020). A historical review of publication bias. Res Synth Methods.

[79] Naci H, Dias S, Ades AE (2014). Industry sponsorship bias in research findings: a network meta-analysis of LDL cholesterol reduction in randomised trials of statins. BMJ.

[80] Owen RK (2020). Multivariate network meta-analysis incorporating class effects. BMC Med Res Methodol.

[81] Papakonstantinou T (2020). In network meta-analysis, most of the information comes from indirect evidence: empirical study. J Clin Epidemiol.

[82] Salanti G, Marinho V, Higgins JP (2009). A case study of multiple-treatments meta-analysis demonstrates that covariates should be considered. J Clin Epidemiol.

[83] Shi C (2018). Node-making processes in network meta-analysis of nonpharmacological interventions should be well planned and reported. J Clin Epidemiol.

[84] Song F (2003). Validity of indirect comparison for estimating efficacy of competing interventions: empirical evidence from published meta-analyses. BMJ.

[85] Swallow E (2020). Causal inference and adjustment for reference-arm risk in indirect treatment comparison meta-analysis. J Compar Effect Res.

[86] Tan SH (2013). Presentational approaches used in the UK for reporting evidence synthesis using indirect and mixed treatment comparisons. J Health Serv Res Policy.

[87] Thorlund K (2013). Why the findings of published multiple treatment comparison meta-analyses of biologic treatments for rheumatoid arthritis are different: an overview of recurrent methodological shortcomings. Ann Rheum Dis.

[88] Tonin FS (2019). Description of network meta-analysis geometry: a metrics design study. PLoS ONE [Electronic Resource].

[89] Lunny C (2023). Knowledge user survey and Delphi process to inform development of a new risk of bias tool to assess systematic reviews with network meta-analysis (RoB NMA tool). BMJ Evid-Based Med.

[90] Whiting P. et al. ROBIS: Tool to assess risk of bias in systematic reviews-Guidance on how to use ROBIS. Available at) (Accessed March 26, 2018) http://www.bristol.ac.uk/media-library/sites/social-community-medicine/robis/robisguidancedocument.pdf.View in Article, 2016.

[91] Cochrane Methods Group, About the Cochrane Methodology Register: http://www.cochranelibrary.com/help/the-cochrane-methodology-register-july-issue-2012.html. 2012, Cochrane.

