# Supplementary material for: Methodological review of NMA bias concepts provides groundwork for the development of a list of concepts for potential inclusion in a new risk of bias tool for network meta-analysis (RoB NMA Tool)
Source: Syst Rev. 2024 Jan 12;13:25. doi: 10.1186/s13643-023-02388-x (PMC10785511; doi:10.1186/s13643-023-02388-x)
Supplement: Supplementary file 1 — Additional file 1: Appendix 1. Steering committee. Appendix 2. Search strategies (July 2020). Appendix 3. Retained concepts. [file 13643_2023_2388_MOESM1_ESM.docx]

**Appendix 1: Steering committee**

• Lunny C, Knowledge Translation Program, Li Ka Shing Knowledge Institute, St. Michael’s Hospital, Unity Health Toronto

• Veroniki A, Knowledge Translation Program, Li Ka Shing Knowledge Institute, St. Michael’s Hospital, Unity Health Toronto

• Dias, S, Centre for Reviews and Dissemination, University of York, York, UK

• Hutton, B, Ottawa Hospital Research Institute, Ottawa, Canada. Ottawa University, School of Epidemiology and Public Health, Ottawa, Canada

• Wright J, Cochrane Hypertension Review Group and the Therapeutics Initiative, University of British Columbia, Canada

• White IR, MRC Clinical Trials Unit at UCL, London, UK

• Whiting P, Population Health Sciences, Bristol Medical School, University of Bristol

• Tricco AC, Knowledge Translation Program, Li Ka Shing Knowledge Institute, St. Michael’s Hospital, Unity Health Toronto, 209 Victoria Street, East Building, Toronto, ON, M5B 1T8, Canada

**Appendix 2: Search strategies (July 2020)**

Ovid MEDLINE (R) and Epub Ahead of Print, In-Process & Other Non-Indexed Citations, Daily and Versions(R) (1946 - )

((network meta-analys?s or NMA or ((indirect or mixed) adj3 comparison)) adj5 (tool? or instrument? or checklist? or check list? or scale? or measure? or assess? or compar*)).ab,ti.

EBM Reviews - Cochrane database of Systematic Reviews (2005 –)

network meta-analys?s or NMA or ((indirect or mixed treatment? or treatment?) adj3 comparison) AND (tool? or instrument? or checklist? or check list? or scale? or assess? or Validity or bias$ or apprais$ or quality)

The EQUATOR Network (http://www.equator-network.org/reportingguidelines/)

Study type: Systematic reviews and contl F “network”

ProQuest Dissertations & Theses Global

TI(Network Meta-Analysis) AND AB(tool)

Cochrane Comparing Multiple Interventions Methods Group https://methods.cochrane.org/cmi/welcome

https://methods.cochrane.org/cmi/relevant-publications-and-resources

(Cochrane Chapter on NMA; and MECIR considerations for NMA – in development)

EBM Reviews - Cochrane Methodology Register (includes Cochrane Colloquium abstracts) (3rd Quarter 2012) (includes Cochrane Colloquium abstracts)

((network meta-analys?s or NMA or ((indirect or mixed) adj3 comparison)) adj5 (tool? or instrument? or checklist? or check list? or scale? or measure? or assess? or compar* or valid$ or invalid or bias$ or apprais$ or quality)).ab,ti.

Scientific Resource Center Methods library of the AHRQ Effective Health Care Program

http://www.refworks.com/refworks2/?site=027181135918800000%2F57381342557464357%2FSRC+Methods+Library

network meta-analysis

International Network of Agencies for Health Technology Assessment: https://www.inahta.org

“network meta-analysis” and “mixed treatment comparison”

Pharmaceutical Benefits Advisory Committee: https://www.pbs.gov.au/info/industry/listing/participants/pbac

“network meta-analysis” and “mixed treatment comparison”

Institut für Qualität und Wirtschaftlichkeit im Gesundheitswesen: https://www.iqwig.de/en/home.2724.html

“network meta-analysis” and “mixed treatment comparison”

European Network for Health Technology Assessment:

https://eunethta.eu/methodology-guidelines/

“network meta-analysis” and “mixed treatment comparison”

Guidelines International Network:

https://g-i-n.net/home

“network meta-analysis”, and “mixed treatment comparison”

International Society for Pharmacoeconomics and Outcomes Research:

https://www.ispor.org/ AND https://tools.ispor.org/peguidelines

“network meta-analysis”, and “mixed treatment comparison”

National Institute for Health and Care Excellence Decision Support Unit:

http://nicedsu.org.uk/multivariate-meta-analysis-tsd

“network meta-analysis”, and “mixed treatment comparison”

Canadian Agency for Drugs and Technologies in Health:

https://www.cadth.ca/

Search study type “reports”, then for “network meta-analysis”, and “mixed treatment comparison”

**Appendix 3: Retained concepts**

Concepts were categorised into the following domains: 3 concepts in network characteristics, 4 concepts in effect modifiers, 13 concepts in statistical synthesis, and 2 concepts in interpretation of the findings and conclusions. These concepts should not be used to assess bias in NMAs as they are preliminary thoughts which will be altered and refined into items based on expert feedback.

| **Domain** | **Concept** |
| --- | --- |
| Network characteristics | 1. Whether all interventions in the network (including comparators) were potentially suitable for all eligible studies |
|  | 2. Whether any interventions were inappropriately excluded from the network (e.g. through eligibility criteria or after seeing the results) |
|  | 3. Whether importantly different intervention strategies were kept as distinct nodes in the network (i.e. whether appropriate groupings were made of interventions -- lumping vs splitting) |
| Effect modifiers | 4. Whether effect-modifying participant characteristics are sufficiently similar across the whole network |
|  | 5. Whether outcomes and timepoints are sufficiently similar across the whole network |
|  | 6. Whether study-level risks of bias are sufficiently similar across the whole network |
|  | 7. Whether other trial characteristics are sufficiently similar across the whole network |
| Statistical synthesis | 8. Whether an appropriate pre-specified approach was used in node making |
|  | 9. Whether a process was used to define nodes in the network (e.g. undertaken independently by two reviewers, following a pre-planned node-making process) |
|  | 10. Whether effect metric(s) for each outcome (e.g. odds ratios, risk ratio) in the network were presented with confidence/credible intervals |
|  | 11. If disconnected networks were connected to perform the analysis, whether methods to do this were appropriate |
|  | 12. Whether methods used to represent multi-arm studies in the dataset and to handle multi-arm studies in the analysis are appropriate |
|  | 13. Whether assumptions made across the network about homogeneity/ heterogeneity of effects within comparisons are appropriate |
|  | 14. Whether a valid approach was used to determine whether there was conflict between direct and indirect sources of evidence on the same comparisons (often called inconsistency or incoherence) |
|  | 15. If inconsistency was detected, then whether methods such as re-evaluation of the choice of scale, effect modification, and similarity of the contributing randomized controlled trials were investigated |
|  | 16. If a Bayesian analysis was conducted, whether the selection of prior distributions was justified |
|  | 17. Whether the analysis appropriately addressed any differences in effect modifiers across different parts of the network |
|  | 18. Whether there was evidence of conflicting results between direct and indirect evidence (often called inconsistency and incoherence in results) |
|  | 19. If there were conflicting results between direct and indirect evidence was this addressed appropriately (e.g. meta-regression, cannot draw conclusions from the results, data extraction errors, redefining the network) |
|  | 20. Evidence that the statistical model, as it was used to get the key results, was not suitable for the data (e.g. from analysis of residuals or information criteria such as DIC) |
| Interpretation of the findings and conclusions | 21. Whether the above questions indicate potential bias in the estimated intervention effects |
|  | 22. Whether the potential bias in the intervention effects is taken into account in the conclusions |

DIC: deviance information criterion
